# Residues of veterinary antibiotics in solid natural and organic fertilizers—method development and sample analysis

**DOI:** 10.1007/s11356-024-33956-w

**Published:** 2024-06-18

**Authors:** Ewelina Patyra, Zbigniew Osiński, Krzysztof Kwiatek

**Affiliations:** Department of Hygiene of Animal Feedingstufs, National Veterinary Research Institute, Pulawy, Poland

**Keywords:** Veterinary antibiotics, Natural fertilizer, Organic fertilizer, UHPL-MS/MS analysis, Method development, Validation

## Abstract

Livestock excrement is used around the world as natural fertilizers or, after processing, as organic fertilizers for crops and grasslands. But due to the presence of veterinary antibiotics in them, they may pose a threat not only to the natural environment, mainly to soil microorganisms, but also to human and animal health. This article describes a method for detecting 21 antibacterial substances in solid natural and organic fertilizers. Antibiotics from fertilizers were extracted with a mixture of acetonitrile and McIlvain-Na_2_EDTA buffer, twice. The extracts were purified by solid phase extraction technique on Strata-X cartridges and analyzed with the use UHPLC-MS/MS technique. The method was validated in accordance with EU Commission Implementing Regulation 2021/808; the obtained recovery ranged from 93.6 to 116.6% (depending on the analytes), and the linearity ranged from 50 to 1000 µg/kg. The developed method was used to analyze 73 samples of solid natural and organic fertilizers. Our research has shown that over 38% of natural fertilizers were contaminated with antibiotics, mainly doxycycline in concentrations reaching several dozen milligrams per kilogram of fertilizers. In the case of processed organic fertilizers, the presence of antibiotics was found in over 37% of the analyzed samples. The research results showed that the developed and validated analytical method may be useful for assessing the presence and content of antibacterial substances in solid natural and organic fertilizers.

## Introduction

Veterinary antibiotics are widely used in animal husbandry worldwide to prevent and treat disease or in some countries still as antibiotic growth promoters. They are administered by injection, as medicated feed, or by dissolving in water (Hou et al. 2015; Zhou et al. 2013; Zhi et al. 2020a, b). Regardless of the route of administration of antibacterial substances to farm animals, these compounds are excreted from the body in amounts ranging from 30 to 90% of the administered initial dose, in unmetabolized form or in the form of active and/or inactive metabolites with feces or urine, which are then used as natural fertilizers for fertilizing arable lands and grassland (Berendsen et al. 2015) or processed (granulation, composting, fermentation) and available on the market as organic fertilizers. Manure, organic fertilizers, and soil improvers are category 2 materials. This means that they may be by-products containing residues of approved substances or contaminants at levels above permitted levels, including antibiotics, sulfonamides, and quinolones, which should be monitored in live animals and products animal origin (Regulation (EC) No. 1069/2009). Natural and organic fertilizers are not subject to any control for the presence of antibacterial substances, and the agricultural use of these fertilizers may cause contamination of the natural environment with these compounds.

Antibiotics are detected unchanged in manure, slurry and poultry litter from farms in concentrations ranging from several μg/kg to several hundred mg/kg. Differences in the levels of antibacterial substances in the above-mentioned fertilizers depend on the animal species, the class of antibiotic, and the geographical location and type of breeding farm. Their content depends on the antibiotic used because some substances, such as amoxicillin or tetracycline, are metabolized only by 10–20%, while others, e.g. sulfamethoxazole, by approximately 85% (Hirsch et al. 1999). The highest concentrations of antibiotics are detected in fertilizers from large-scale farms compared to small family farms (Zhi et al. 2020a, b). In addition, detection rates and antibiotic concentrations are usually higher in pigs’ fertilizers than poultry and cattle fertilizers. This is mainly due to the fact that antibiotics are administered in higher doses and more frequently to pigs than to other farm animals (Xin et al. 2016). In recent years, researchers have published several papers demonstrating that antibiotics in feces derived from slaughtered animals are present in high concentrations.

Wolters et al. (2016) examined derived manure from eight fattening farms and six breeding pig farms. In the material examined, the authors found 11 different antibiotics belonging to up to six classes. Antibiotic residue analysis showed a maximum tetracycline concentration reaching up to 300 mg/kg dry matter (DM) in pig manure (Wolters et al. 2016). Martinez-Carballo and colleagues examined pig feces in Austria, in which they found the presence of antibiotics from the tetracycline group in amounts of several dozen milligrams per kilogram of feces (chlorotetracycline—46 mg/kg, oxytetracycline—29 mg/kg, and tetracycline—23 mg/kg) (Martinez-Carballo et al. 2007). Researchers from China were analyzing fertilizers from chicken, in which they found high concentrations of enrofloxacin and norfloxacin of 1420 mg/kg and 225 mg/kg, respectively (Zhao et al. 2010).

The literature describes methods enabling the analysis of antibiotics in natural fertilizers using liquid chromatography techniques with various detectors: fluorescence, UV, single mass spectrometer, or tandem mass spectrometry (Berendsen et al. 2015; Haller et al. 2002; Jansen et al. 2019; Karci and Balcioglu 2009; Martinez-Carballo et al. 2007; Xian-Gang et al. 2008; Wallace and Aga 2016; Zheng et al. 2021). However, these methods most often involve the analysis of natural fertilizers such as poultry, pig, or cattle excrement (Berendsen et al. 2015; Haller et al. 2002; Jansen et al. 2019; Xian-Gang et al. 2008; Zheng et al. 2021). The extraction and chromatographic analysis method we have developed allows the analysis not only of solid natural fertilizers, such as animal excrement, but also of solid organic fertilizers subjected to processing processes, such as drying and granulation. Due to the lack of laboratory tests on the presence and content of antibiotics in solid natural and organic fertilizers, the aim of this work was to develop and validate an analytical method for the quantitative determination of antibiotics from various chemical groups in one analytical course. The study compared different antibiotic extraction solvents and different solid phase extraction cartridges. After selecting the optimal preparation stage, the samples were analyzed using the UHPLC-MS/MS technique. The developed method was used to analyze real samples of solid natural fertilizers from pigs, poultry, and cattle and commercially available solid organic fertilizers produced from animal by-products.

## Materials and methods

### Chemicals and reagents

HPLC-grade methanol and acetonitrile were purchased from J.T. Baker (Deventer, the Netherlands). Citric acid and formic acid (purity > 99% for analysis) were obtained from Acros Organics (Geel, Belgium). Disodium hydrogen phosphate was from Chempur (Piekary Śląskie, Poland) and disodium ethylenediamine tetraacetate (Na_2_EDTA) was form Sigma Aldrich (CA, MO, USA). Water was purified using a Milli-Q water system from Millipore (Billerica, Ma, USA). Four SPE cartridges were tested: Oasis HLB (3 mL, 60 mg) from Waters (Milliford, MA, USA), Strata-S (6 mL, 200 mg), Strata-SAX (12 mL, 500 mg), and Strata-XCW (3 mL, 300 mg) from Phenomenex (Torrance, CA, USA).

All target veterinary antibiotics (VAs) and internal standards (IS) were purchased from Dr. Ehrenstorfer Gmbh (Augsburg, Germany). The 21 target antibiotics belonged to 6 classes: tetracyclines (TCs): oxytetracycline (OXT), epi-oxytetracycline (epi-OXT), tetracycline (TC), chlortetracycline (CTC), epi-chlotetracycline (epi-CTC), doxycycline (DC), and demeclocycline (DMC; IS); sulfonamides (SAs): sulfaguanidine (SGD), sulfadiazine (SDZ), sulfamerazine (SMR), sulfamethazine (SMZ), sulfamethoxazole (SMX), and sulfadiazine-^13^C_6_ (SDZ-^13^C_6_; IS); fluoroquinolones (FQs): ciprofloxacin (CIP), enrofloxacin (ENR), sarafloxacin (SAR), flumequine (FLU), and norfloxacin (NOR; IS); macrolides (MAs): tylosin (TYL), spiramycin (SPIR), and erythromycin (ERM; IS); pleuromutilin (PLM): tiamulin (TIAM) and valnelmulin (VAL); and lincosamides: lincomycin (LINCO) and lincomycin-d_3_ (LINCO-d_3_; IS).

McIlvaine Na_2_EDTA buffer was prepared by dissolving 11.406 g Na_2_EDTA in 115.65 mL 0.2 M phosphate buffer and 184.65 mL 0.1 M citric acid. The pH was adjusted to 4.0. Four SPE cartridges were tested OASIS HLB (60 mg, 3 mL) from Waters (Milford, MA, USA) and Strata-X (200 mg, 6 mL), and Strata-X-CW (300 mg, 3 mL), Stata-SAX (500 mg, 12 mL) from Phenomenex (Torrance, CA, USA). An SPE manifold (J.T. Baker, PA, USA) and a pump as a vacuum source were used.

### Preparation of standard solutions

Stock standard solutions 1 mg/mL of OXT, epi-OXT, TC, CTC, epi-CTC, DC, SGD, SMR, SMZ, SXZ, TRIM, ENR, SAR, LICO, TIAM, TYL, SPIR, VAL and internal standards of ERT, DMC and LINCO-d_3_ were prepared by dissolving 5 mg of individual compounds in 5 mL of methanol. SDZ, SDZ-^13^C_6_, FLU, and NOR were dissolved in acetonitrile and CIP was dissolving in mixture of methanol and 1 M sodium hydroxide (99:1; v/v). All standard solutions were stored in volumetric flasks at – 18 °C for 6 months. All VAs and IS working solutions of 10 µg/mL were prepared by diluting the stock solutions in methanol and stored in amber volumetric flask at – 18 °C for less than 1 month.

### Instrument analysis

Quantification of antibiotics were performed by UHPLC-MS/MS consisted of an Exion LC with a SCIEX Triple Quad 5500 + System (SCIEX, Framingham, MA, USA). Kinetex C18 column (2.1 mm × 75 mm; 2.6 µm) was employed to separate the target compounds at 35 °C and the flow rate of mobile phase was 0.25 mL/min. The mobile phase consisted of 0.1% formic acid in Milli-Q water (A) and 0.1% formic acid in acetonitrile (B), and the gradient elution was as follows: 0–2 min 5% B, 2–10 min 5–15% B, 10–12 min 15–20% B, 12–15 min 20–50% B, 15–16 min 50–70% B, 16–17 min 70–100%, 17–18 100–5% B, and 18–21 min 5% B. The injection volume was 10 µL.

The criteria to identify the different antibiotics and their active metabolites were detecting the masses of the precursor and fragments ions. MS/MS detection was performed under the multiple-reaction monitoring (MRM) using positive electrospray ionization mode (ESI +) for all antibiotics. In addition, different parameters were used for the operation of the mass detector. Detailed parameters of MS/MS are listed in Tables 1 and 2. The chromatographic integration of the samples was performed using Sciex OS MQ software version 2.1.6 (SCIEX, Framingham, MA, USA).

**Table 1:** Operating parameters of the MS/MS detector

Parameters	Analytical conditions
Curtain gas	30 psi
Collision gas	10 psi
Ion spray voltage	4500 V
Temperature	400 °C
Ion source gas 1	40 psi
Ion source gas 2	40 psi
Entrance potential	10

**Table 2:** Transitions and optimal conditions used for MS/MS analysis

No	Analyte	Precursor ion Q1	Product ion Q3	DP	CE	CXP	EP	Dwell time [ms]
Tetracyclines								
1	Oxytetracycline 1	461.0	426.2	116	27	22	10	150
	Oxytetracycline 2	461.0	444.2	116	19	12	10	150
2	Tetracycline 1	445.0	410.0	50	27	15	10	150
	Tetracycyline 2	445.0	427.0	60	20	15	10	150
3	Chlorotetracycline 1	479.0	444.2	126	31	22	10	150
	Chlorotetracycline 2	479.0	462.2	126	25	20	10	150
4	Doxycycline 1	445.0	428.1	70	27	12	10	150
	Doxycycline 2	445.0	410.0	70	33	12	10	150
5	Epi-oxytetarcycline 1	461.0	426.0	27	116	22	10	150
	Epi-oxytetracycline 2	461.0	444.2	19	116	12	10	150
6	Epi- chlortetracycline1	479.0	444.2	31	126	22	10	150
	Epi-chlortetracycline 2	479.0	462.2	25	126	20	10	150
IS	Demeclocycline	465.0	448.1	124	25	16	10	150
Fluoroquinolone								
7	Ciprofloxacin 1	332.0	314.0	79	29	13	10	100
	Ciprofloxacin 2	332.0	245.0	61	35	13	10	100
8	Enrofloxacin 1	360.0	342.0	80	30	24	10	100
	Enrofloxacin 2	360.0	286.0	80	36	24	10	100
9	Sarafloxacin 1	386.0	368.0	29	31	13	10	100
	Sarafloxacin 2	386.0	299.0	39	45	13	10	100
10	Flumequine 1	262.0	202.0	63	45	14	10	150
	Fumequine 2	262.0	174.0	60	51	14	10	150
IS	Norfloxacin	319.1	151.1	104	17	18	10	150
Sulfonamides								
11	Sulfaguandine 1	215.0	156.0	86	21	18	10	150
	Sulfaguanidine 2	215.0	92.0	86	33	12	10	150
12	Sulfadiazine 1	251.0	156.0	31	23	18	10	150
	Sulfadiazine2	251.0	108.0	55	32	15	10	150
13	Sulfamerazine1	265.0	156.0	35	25	12	10	150
	Sulfamerazine 2	265.0	92.0	44	40	12	10	150
14	Sulfamethazine 1	279.0	186.0	14	25	12	10	150
	Sulfamethazine 2	279.0	124.0	13	26	12	10	150
15	Sulfamethoxazole 1	254.0	156.0	56	23	18	10	150
	Sulfamethoxazole 2	254.0	108.0	53	33	18	10	150
16	Trimethoprim 1	291.0	230.1	181	33	12	10	150
	Terimethoprim 2	291.0	261.1	181	35	14	10	150
IS	Sulfadiazine-^13^C_6_	257.0	162.0	89	23	16	10	150
Lincosamides								
17.	Lincomycin 1	407.0	126.0	65	39	13	10	150
	Lincomycin 2	407.0	359.0	81	27	13	10	150
IS	Lincomycin-d_3_	410.1	129.2	129	37	14	10	150
Macrolides								
18	Spiramycin 1	422.0	174.0	126	29	16	10	150
	Spiramycin 2	422.0	101.0	126	25	12	10	150
19	Tylosin 1	916.0	174.1	139	51	22	10	150
	Tylosin 2	916.0	772.4	125	43	20	10	150
IS	Erytrhomycin	734.4	576.4	157	41	12	10	150
Pleuromutilin								
20	Tiamulin 1	494.0	192.0	114	29	10	10	150
	Tiamulin 2	494.0	119.0	135	55	12	10	150
21	Valnemulin 1	565.2	263.1	141	25	16	10	150
	Valnemulin 2	565.2	164. 1	141	25	10	10	150

### Method development

Due to the fact that the analytical matrices selected for research, such as natural and organic fertilizers, are complex analytical matrices, developing an appropriate extraction method for all antibacterial substances listed in Table 3 turned out to be a time-consuming step. The extraction method was optimized for samples fortified with analytes at a level of 100 µg/kg for solid fertilizers.

**Table 3:** Validation results of the LC–MS/MS method for determining antibacterial substances in solid natural fertilizers (pig feces)

Validation parameters		Analyte									
		OTC	Epi-OTC	TC	CTC	Epi-CTC	DC	CIP	ENR	SAR	FLU
Selectivity		No interference									
LOD (µg/kg)		15.4	25.1	22.9	20.1	22.6	31.1	20.9	29.0	21.7	24.0
LOQ (µg/kg)		24.3	43.2	40.0	31.0	37.4	45.5	43.6	62.0	41.2	45.1
CCα (µg/kg)		62.7	63.7	61.0	56.8	67.6	68.9	60.8	66.5	58.3	51.2
CCβ (µg/kg)		74.3	76.8	74.1	68.3	84.0	90.1	76.3	97.0	72.1	62.7
Repeatability (CV %)	50 µg/kg	8.7	15.0	5.4	12.6	15.0	12.9	13.9	17.4	17.1	14.9
	500 µg/kg	8.7	11.3	9.2	12.1	11.3	6.1	14.0	15.7	10.0	15.8
	1000 µg/kg	6.6	6.3	6.7	10.0	6.3	9.4	12.5	15.9	13.0	14.4
Reproducibility (CV %)	50 µg/kg	10.1	16.1	10.7	14.5	16.1	17.6	11.7	18.4	13.6	12.0
	500 µg/kg	14.0	11.4	15.5	14.5	11.4	15.0	14.3	21.4	13.7	15.5
	1000 µg/kg	8.4	9.9	9.0	8.9	6.3	10.3	11.9	18.8	16.8	14.0
Recovery (%)	50 µg/kg	105.4	110.4	102.6	108.4	103.4	101.7	112.4	103.9	112.4	114.2
	500 µg/kg	93.6	102.5	94.2	104.0	99.4	99.6	97.5	97.5	93.9	98.9
	1000 µg/kg	98.9	103.6	99.8	107.2	106.8	110.7	105.1	100.7	103.2	98.6
Uncertainty (U %)	50 µg/kg	33.0	35.0	25.0	30.0	33.5	35.2	28.0	37.0	34.0	30.1
	500 µg/kg	29.0	27.3	33.0	29.2	31.0	32.0	28.4	35.0	31.0	28.7
	1000 µg/kg	27.8	24.0	24.0	18.0	28.4	23.1	26.5	35.7	30.5	25.3

Validation parameters		Analyte										
		SGD	SDZ	SMR	SMZ	SMK	TRIM	LINKO	TIAM	TYL	SPIR	VAL
Selectivity		No interference										
LOD (µg/kg)		36.6	14.0	16.5	19.3	21.0	9.3	13.4	13.4	12.4	15.5	19.7
LOQ (µg/kg)		48.9	27.5	39.0	46.5	49.0	18.8	27.5	24.8	22.3	27.0	33.1
CCα (µg/kg)		68.9	58.4	52.2	57.7	54.3	55.1	64.8	63.3	74.5	60.0	65.0
CCβ (µg/kg)		82.3	64.4	59.1	64.3	71.2	62.7	82.3	76.2	94.2	81.1	84.2
Repeatability (CV %)	50 µg/kg	18.9	10.3	7.7	10.8	10.8	18.2	8.2	14.2	17.0	15.7	16.1
	500 µg/kg	15.3	11.4	11.3	11.3	8.7	11.5	14.4	8.7	12.1	10.8	13.8
	1000 µg/kg	12.3	5.7	6.8	8.3	10.6	5.8	10.3	8.1	6.0	16.1	8.6
Reproducibility (CV %)	50 µg/kg	19.1	10.8	14.3	10.1	10.6	14.4	14.3	9.8	17.5	13.4	16.4
	500 µg/kg	13.5	17.8	11.1	10.8	18.3	12.5	13.0	12.6	10.9	18.7	14.4
	1000 µg/kg	10.3	12.6	10.5	11.5	9.8	6.9	12.5	12.6	8.8	12.7	12.0
Recovery (%)	50 µg/kg	110.0	103.6	113.8	116.6	11.8	98.7	108.0	110.5	107.9	105.1	104.4
	500 µg/kg	99.6	102.4	101.3	98.7	101.3	99.4	9.8	95.5	95.4	100.7	97.7
	1000 µg/kg	106.7	103.3	104.0	107.1	102.0	94.8	96.2	102.5	96.0	101.0	105.5
Uncertainty (U %)	50 µg/kg	36.2	28.6	28.5	24.5	22.0	30.0	25.0	27.6	35.0	26.7	33.0
	500 µg/kg	30.4	25.1	23.0	22.3	27.7	24.8	22.1	25.0	30.0	34.1	27.1
	1000 µg/kg	29.5	24.4	18.8	22.0	21.9	18.5	15.6	24.3	28.8	26.6	22.9

### Extraction experiments

Twenty-one compounds were selected from six different antibiotics classes: the tetracyclines OXT, epi-OXT, TC, CTC, epi-CTC, and DC; fluoroquinolones CIP, ENR, SAR, and FLU, the sulfonamides SGD, SDZ, SMR, SMZ, SXZ, and TRIM; the macrolides TYL and SPIR; pleuromutilin TIAM and VAL; and lincosamide LINCO. Different physico-chemical properties characterize the mentioned antibiotics; therefore, experiments had to be carried out using various extraction mixtures that would allow for good extraction of all the mentioned analytes from natural and organic fertilizers. In addition, due to the complexity of the analytical matrix, it was necessary to select an appropriate technique for purifying the obtained extracts to ensure detection and good recovery of all analyzed antibiotics.

#### Experiment I

**Fig. 1:**
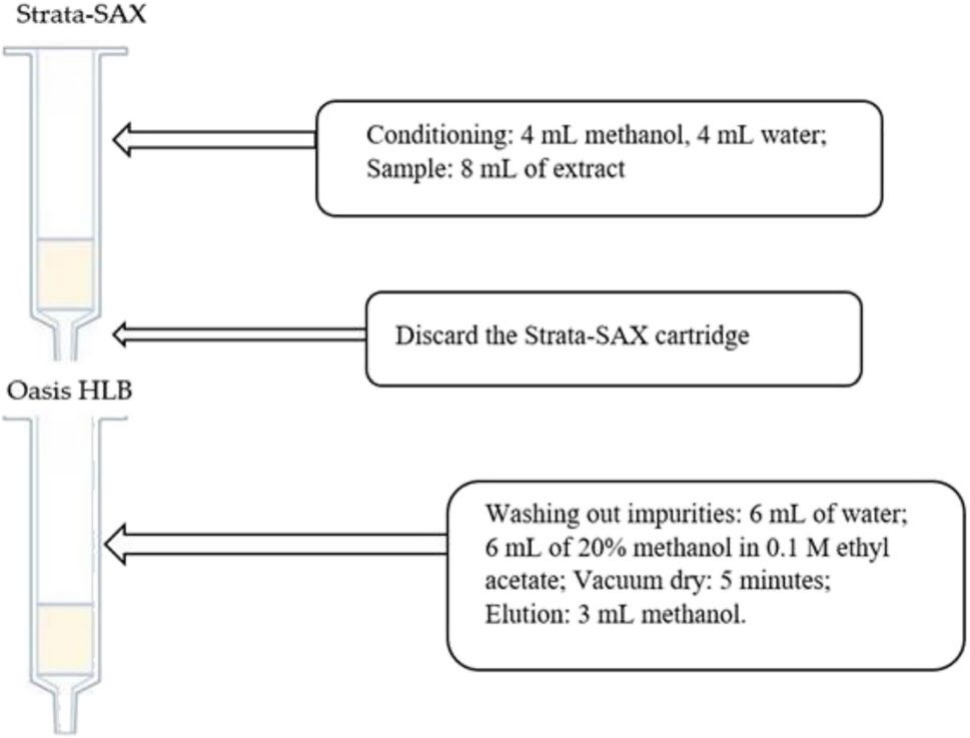
Purification of the extract using “tandem” solid phase extraction

Two- and 5-g samples of solid fertilizer were extracted with 25 mL of a mixture of McIlvaine-Na_2_EDTA buffer at pH = 7 and acetonitrile in a ratio of 23:2; v/v. The samples were shaken and centrifuged, then the extract was filtered through a cellulose filter, and acidified with 85% orthophosphoric acid using 50 µL of orthophosphoric acid for each 5 mL of extract. After this stage of work, the extract was purified using “tandem” solid-phase extraction according to the scheme below (Fig. 1).

The obtained eluate was evaporated in a stream of nitrogen, and the precipitate was dissolved in 500 µL of 0.1% formic acid in water and additionally filtered through a PVDF syringe filter with a diameter of 13 mm and a pore size of 0.22 µm.

#### Experiment II

Two- and 5-g solid fertilizer samples were extracted with 25 mL of McIlvaine-Na_2_EDTA buffer with pH = 4. The samples were shaken and centrifuged. The extract was purified by SPE using Strata-XCW cartridges (3 mL, 100 mg). The cartridges were conditioned with 5 mL of methanol, 5 mL of water, then 9 mL of the extract was placed on the cartridge. The impurities were washed out with 6 mL of water, 6 mL of methanol, and 3 mL of acetonitrile. The cartridges were dried under vacuum for 5 min, and the antibacterial substances were eluted from the columns with 3 mL of 2% formic acid in methanol. The eluate was evaporated in a stream of nitrogen, and the obtained precipitate was dissolved in 0.1% formic acid in water and additionally filtered through a PVDF syringe filter with a diameter of 13 mm and a pore size of 0.22 µm.

#### Experiment III

Two- and 5-g samples of solid fertilizer were extracted with 25 mL of a mixture of McIlvaine-Na_2_EDTA buffer at pH = 4 and acetonitrile in a ratio of 23:2; v/v. The samples were shaken and centrifuged. The extract was purified by SPE using Strata-X cartridges (6 mL, 200 mg). The cartridges were conditioned with 6 mL of methanol and 6 mL of water. After conditioning, 12 mL of extract was dosed onto the cartridges, the impurities were washed out with 12 mL of water, and then the cartridges were dried for 10 min under vacuum. Antibacterial substances were eluted from the cartridges with 3 mL of methanol. The eluate was evaporated in a stream of nitrogen, and the precipitate was dissolved in 500 µL of 0.1% formic acid in first-class water and filtered through a PVDF syringe filter with a diameter of 13 mm and a pore size of 0.22 µm.

#### Experiment IV

A 2-g solid fertilizer sample was extracted with a mixture of McIlvaine-Na_2_EDTA buffer at pH = 4, acetonitrile, and methanol in the proportion of 3:3.75:1.25 v/v/v or for 5-g samples in the ratio 6:7.5:2.5 v/v/v. The samples were shaken and centrifuged. To reduce the content of organic reagents such as acetonitrile and methanol, the obtained extract was diluted in water by adding 6 mL of extract to 25 mL of water. The extracts were purified by SPE using Strata-X cartridges (6 mL, 200 mg). The cartridges were conditioned with 6 mL of methanol and 6 mL of water. After conditioning, 18 mL of extract was dosed onto the cartridges, the impurities were washed out with 12 mL of water, and then the cartridges were dried for 10 min under vacuum. Antibacterial substances were eluted from the columns with 3 mL of methanol. The eluate was evaporated under a stream of nitrogen, and the precipitate was dissolved in 500 μL of 0.1% formic acid in water and filtered through a 13-mm PVDF syringe filter with a pore size of 0.22 µm.

### Method validation

Validation of the method was carried out following the guidelines set out in the EU Commission Implementing Regulation 2021/808 of March 22, 2021, on the performance of analytical methods for residues of pharmacologically active substances used in food-producing animals and the interpretation of the results, as well as on the methods used for sampling and repealing Decisions 2002/657/EC and 98/179/EC.

### Linearity, selectivity, LOD, and LOQ

The working range of the method was determined by preparing calibration curves for fortified samples. For this purpose, blank samples were fortified with antibacterial substances at seven adopted concentration levels (0, 50, 100, 250, 500, 750, and 1000 µg/kg). The regression coefficient (*R*^2^), slope (*a*), and shift (*b*) were calculated for the linear regression equation of the type *y* = *ax* + *b*. Then, curves were determined for fortified samples based on the relationship between concentration and signal size or the ratio of the analyte signal to the corresponding signal of the internal standard. A satisfactory linearity of the calibration curve for each analyte was assumed based on the coefficient of determination (*R*^2^) value higher than 0.98 for quantification. The limit of detection and limit of quantification were evaluated based on the signal-to-noise ratio (3 for LOD and 10 for LOQ). To determine the selectivity of the method, 20 feces samples were analyzed to check the possible presence of interferences resulting from the endogenous matrix composition in the retention times of the monitored antibiotics.

### Recovery, repeatability, and within-laboratory reproducibility

To determine the recovery and repeatability of the analyses, blank samples were fortified at three concentration levels: 50, 500, and 1000 µg/kg (six samples for each levels). The recovery was calculated based on the results obtained when determining repeatability. Recovery is the percentage of the actual concentration of a substance contained in a sample determined during the analytical process, according to the following equation: % recovery = 100 × measured content/fortification level. Within-laboratory reproducibility was assessed by spiking two other sets of blank solid manure (pig feces) samples at the same concentrations as for repeatability and analyzing them on different days with the same instrument. The mean value (*x*), standard deviation (SD), and coefficient of variation (CV) were calculated for each fortification level.

### Decision limit and detection capability

The decision limit (CCα) was calculated with a statistical certainty of 1 – *α* (*α* = 1%), whereas detection capability (CCβ) was calculated with a statistical certainty of 1 – *β*. Detection capability was calculated as decision limit plus 1.64 times the corresponding standard deviation (*β* = 5%). Selectivity of the method was tested by analyzing 20 blank feces samples to verify the absence of potential interfering endogenous compounds at the target analyte retention times.

### Uncertainty

The uncertainty components are expressed as standard uncertainty, which is measured by the standard deviation. The total uncertainty was expressed as the combined standard uncertainty (uc), and the expanded uncertainty (*U*) was assumed as the product of the combined standard uncertainty and the coverage factor *k* = 2 for the adopted significance level *α* = 0.05 according to the formula: *U* = *k* * *u*_*c*_(*y*).

### Antibiotics monitoring in solid manures and commercial organic fertilizers

Samples of solid natural and organic fertilizers were collected from pig, poultry, and cattle farms and organic fertilizer producers in Poland. In general, the developed method analyzed 24 samples of commercial organic fertilizers produced with animal by-products and 49 samples of solid natural fertilizers, including 41 samples from pigs, 7 samples from poultry, and 1 sample from beef cattle. After delivery to the laboratory, the samples were stored in plastic containers at a temperature of – 18 °C to avoid degradation of antibiotics. Before the analysis day, the samples were slowly thawed in a refrigerator at temperatures of + 2 to 8 °C.

## Results and discussion

### Instrumental conditions

The selected compounds were detected with a mass spectrometer (MS). All the compounds in the study were sensitive in electrospray ionization (ESI) positive mode. The protonated ion was present as the base peak of the MS spectrum and selected as the precursor ion, and two transitions of the precursor ion were selected for quantification and confirmation by optimizing the collision energy.

Chromatographic conditions were optimized to improve separation, sensitivity, and selectivity taking into account the compound investigated. The mobile phase optimization was necessary to obtain satisfactory response for the different compounds at the different concentration levels and for each type of matrix selected (natural and organic solid fertilizer). For the analysis of antibacterial substances from fertilizers and soil matrices, scientists most often use a mobile phase consisting of ammonium acetate, formic acid, and ammonium formate in water in combination with methanol or acetonitrile (with or without formic acid, formate ammonium, or ammonium acetate) (Berendsen et al. 2015; Jansen et al. 2019; Ho et al. 2014; Li et al. 2015; Haller et al. 2002; Wallace and Aga 2016; Wu et al. 2014). In the case of our research, the most favorable mobile phase for separation was a mixture of 0.1% formic acid in water combined with 0.1% formic acid in acetonitrile. The separation of pharmaceuticals belonging to different chemical groups requires the appropriate selection of chromatographic columns in order to obtain the appropriate shape, separation, and peak area. For this purpose, researchers used chromatographic columns such as such as Nucleosil C18 HD, Kinetex C18, Genesis C18, and ACQUITY UPLC BEH C18 (Berendsen et al. 2015; Jansen et al. 2019; Martinez-Carballo et al. 2007; Blackwell et al. 2004; Li et al. 2015; Wu et al. 2014; Hu et al. 2010). However, all chromatographic columns used were filled with octadecyl (C18). In our work, we tested two Kinetex C18 chromatographic columns (both from Phenomenex) differing in length and grain diameter of the filling: 75 mm × 2.1 mm, 2.6 µm and 100 × 4.6 mm, 5 µm. Ultimately, a shorter Kinetex C18 75 × 2.1 mm, 2.6 µm column was selected for the development of the method, on which a satisfactory separation of all 21 analytes was achieved.

### Extraction experiments

Analyzing pharmaceuticals in animal feces, manure, and organic fertilizers can be difficult because it is a complex matrix with a high organic matter content such as undigested food remains (fiber, proteins, and fats), pigments (bilirubin and biliverdin), significant amounts of nitrogen and phosphorus compounds, enzymes, bacteria, and dead lining cells of intestinal walls. In addition, the desire to analyze many antibiotics belonging to different chemical groups and exhibiting different physico-chemical properties requires a lot of effort from the analyst to optimize the extraction mixture and select the appropriate purification method. Therefore, as part of the presented work, we checked four different extraction mixtures (described in other scientific studies), different fertilizer sample weights, and solid-phase extraction cartridges with different sorbents and from different manufacturers.

Based on the tested extraction mixtures, sample weights and SPE cartridges described in the “Extraction experiments” section, it was shown that the use of “tandem” solid phase extraction (experiment I) using combined Strata-SAX and Oasis HLB cartridges may be useful for the analysis of tiamulin, tylosin, trimethoprim, oxytetracycline, epi-oxytetracycline, tetracycline, chlortetracycline, epi-chlortetarcycline, doxycycline, ciprofloxacin, enrofloxacin, sulfamerazine, and sulfamethazine—a total of 11 compounds out of 21 antibacterial substances selected for testing. In addition, the purification technique used, combining two SPE cartridges, did not allow for the detection of 10 analytes and increased the limit of detection of the analytes selected for testing. The presented method was described by Blackwell et al. (2004) and is used for the quantitative analysis of oxtetracycline, sulfachloropyridazine, and tylosin in soil and slurry samples in the concentration range from 0.2 to 5 mg/kg.

In the described extraction and purification procedure for experiment II, the method described by Patyra and Kwiatek (2017) was used, which concerns the analysis of tetracyclines in feed using the LC–MS technique. For this extraction method, McIlvaine-Na2EDTA buffer with pH = 4 was used. McIlvaine-Na2EDTA buffer is often used alone or in combination with methanol or acetonitrile for the extraction of tetracycline antibiotics from biological matrices and feeds. Better results for the analyzed antibiotics were obtained for 5 g of fertilizer samples compared to 2 g of samples. Strata-XCW cartridges were used to purify the extract, and satisfactory results were obtained for trimethoprim, tiamulin, tylosin and ciprofloxacin, enrofloxacin, sarafloxacin and oxytetracycline, tetracycline, chlortetracycline, doxycycline, and their epimeric forms: epi-oxytetracycline and epi-chloroteracycline. The tested method was unsuitable for the sulfonamides selected for testing, lincomycin, spiramycin, valnemulin, and flumequine. The best results were obtained for the described experiments III and IV of extraction and purification of antibacterial substances from solid fertilizers because all analyzed antibacterial substances were observed in the chromatograms. However, both presented methods still had some shortcomings that needed to be improved to obtain the best possible parameters for the developed method. Preparation of fertilizer samples according to the extraction procedure described as “experiment IV” required additional dilution of the extract in water in order to be able to clean-up and concentrate the obtained extract on reverse-phase polymer cartridges, Strata-X. This was due to the extraction mixture used, which included McIlvaine-NA_2_EDTA buffer and acetonitrile and methanol in the proportions of 3 mL/3.75 mL/1.25 mL (or 6 mL/7.5 mL/2.5 mL for a sample weighing 5 g). Direct application of an extract containing approximately 62.5% of the organic mixture to a Strata-X cartridge would cause the analytes to pass through the cartridge along with the dosed extract.

**Fig. 2:**
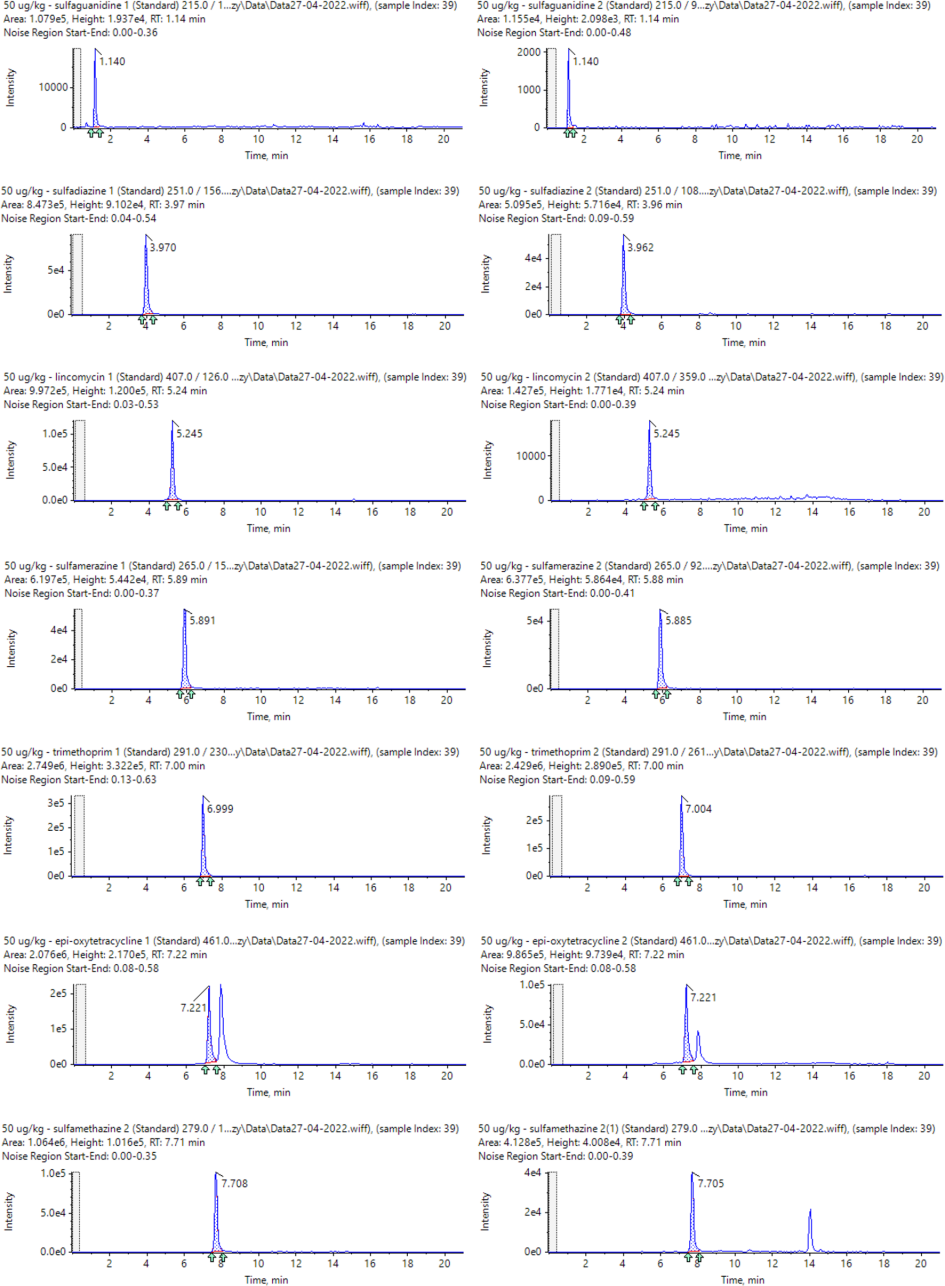

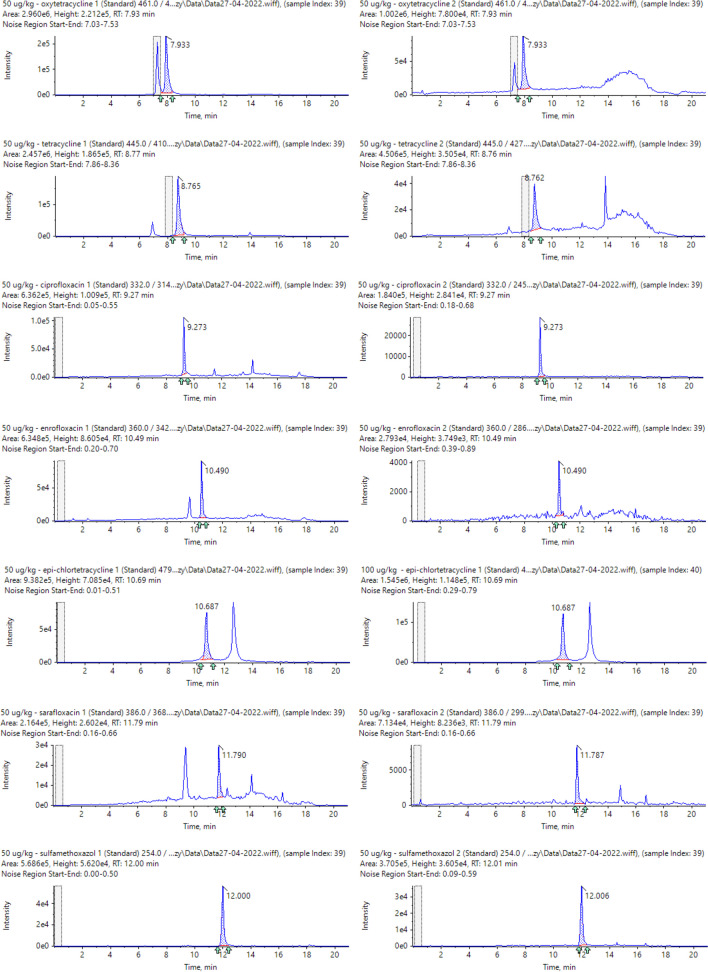

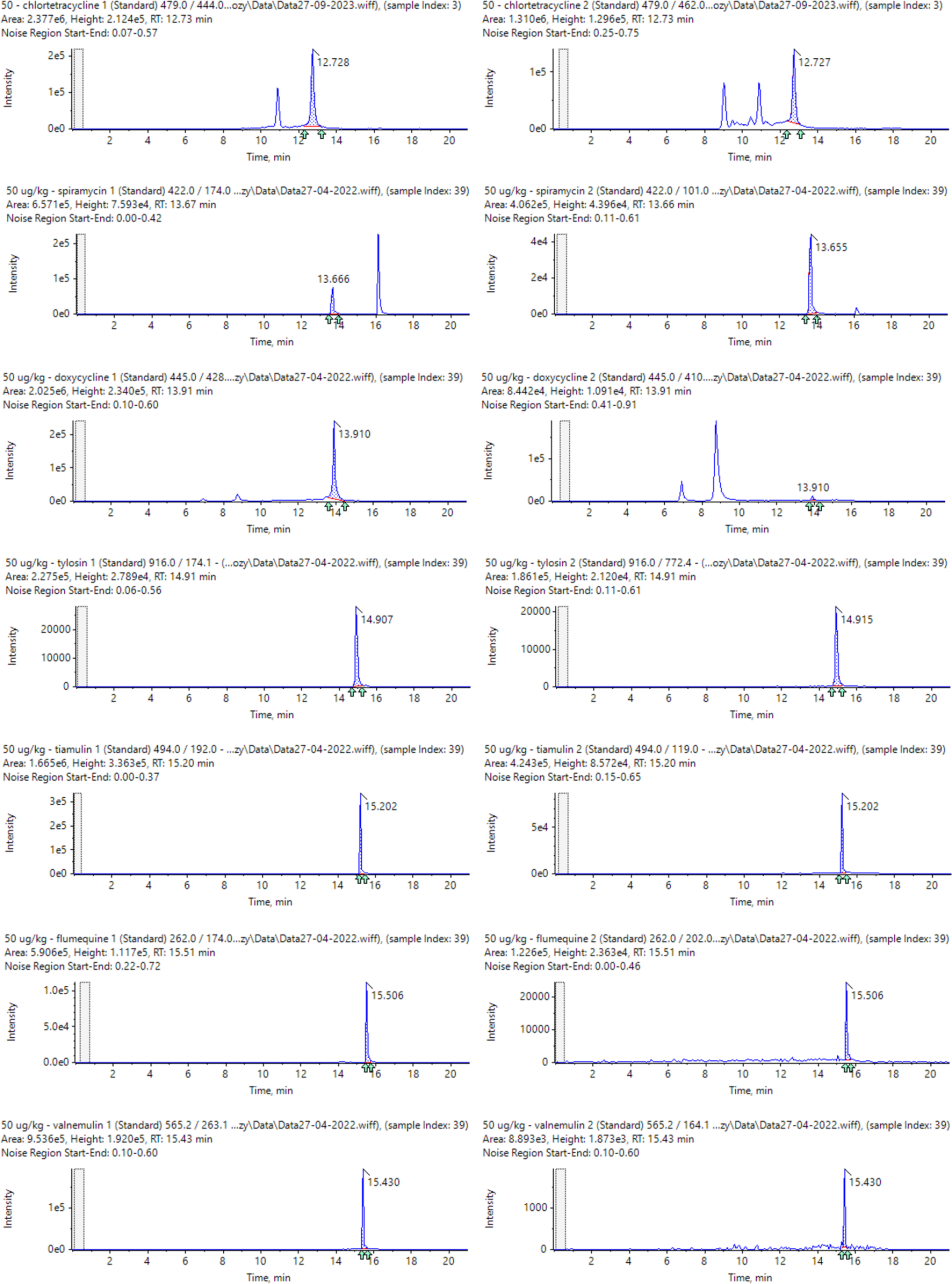
MRM chromatograms for all analyzing antibiotics at a concentration 50 µg/kg

In the case of extraction of antibacterial substances from solid natural fertilizers, better results were obtained for a 2-g sample and double extraction using a mixture of McIlvaine-Na_2_EDTA buffer with pH = 4 and acetonitrile in a ratio of 23:2; v/v and then the McIlvaine-Na_2_EDTA buffer itself. The combined extracts were further degreased with n-hexane and purified on Strata-X cartridges (6 mL, 200 mg) (experiment III). The extraction and purification method used allowed for the detection and quantitative determination of all 21 antibiotics belonging to 6 chemical classes. MRM chromatograms for all analyzing antibiotics at a concentration 50 µg/kg are shown in Fig. 2.

### Method validation

Validation of the developed method was carried out in accordance with Commission Implementing Regulation (EU) 2021/808 of March 22, 2021, on the performance of analytical methods for residues of pharmacologically active substances used in food-producing animals and the interpretation of the results, as well as on the methods used for sampling and repealing Decisions 2002/657/EC and 98/179/EC. The linearity and working range of the method were checked in the concentration range from 50 to 1000 µg/kg for all tested antibiotics in solid fertilizers. A calibration curve showing the ratio of the analyte signal to the corresponding internal standard signal or the dependence of the peak area on the analyte concentration was plotted at seven points, taking into account the blank sample. This curve was used to calculate the concentration of antibiotics in fortified samples. The test results of three series of samples fortified to concentrations of 50, 500, and 1000 µg/kg for solid fertilizers were the basis for determining such validation parameters of the procedure as repeatability, intra-laboratory reproducibility, recovery, decision limit, detection capability, limit of detection, and limit of quantification and uncertainty.

In the presented method, the recovery values of the spiked samples were in the range of 93.6–116.6% for all analyzed antibacterial substances. The intra-day and inter-day precisions of the methods were evaluated at three concentration levels (50, 500, and 1000 μg/kg). For this purpose, six spiked samples at each level were prepared and analyzed. This procedure was repeated for 3 days in order to determine the inter-day precision. The repeatability for the target analytes was lower than 19. The within-laboratory reproducibility was lower than 21% for all analyzing antibacterial substances at all spiking levels. The LOD for the all analyzing VAs in solid natural and organic fertilizers was 12.4–36.6 μg/kg. The LOQ was 22.3–62.0 μg/kg for all analyzing antibacterial substances. The results of the experiments performed are presented in Table 3.

### Real-sample analysis

Seventy-three samples of solid natural fertilizers (pig and cattle manure and poultry droppings) and organic fertilizers produced using animal by-products (such as cattle, horse, sheep, and chicken manure) were analyzed in the study. The results showed that in the case of samples from farmed poultry, only one sample contained the presence of antibacterial substances—sulfamethoxazole at a concentration below 2 mg/kg. Manure from beef cattle analyzed for antibacterial substances were free of antibiotics. The most contaminated with antibiotics was solid manure from pigs from large-scale farms. The most frequently detected antibiotics were tetracyclines, mainly doxycycline. The presence of doxycycline was confirmed in 15 samples of natural fertilizers. Doxycycline has been found at concentrations ranging from 103.0 to over 57,000.0 µg/kg. The tested solid manure from pigs also contained the presence of sulfamethoxazole (in four samples), oxytetracycline and epi-oxytetracycline (in three samples), and tiamulin in two samples. Moreover, the data obtained indicate that two or even three antibiotics were used one after another in the animals from which the material was collected. Two manure samples from piglets aged 5 and 8 weeks revealed the presence of three different antibiotics: doxycycline, oxytetracycline, and tiamulin. This indicates the intensive, perhaps irrational, use of antibiotics in young animals to prevent the development of bacterial infections. Based on the results obtained, it can be concluded that over 38.77% of the tested samples of natural fertilizers, mainly from pigs, were contaminated with antibiotics. The results of the analysis of natural fertilizers showed that tetracycline antibiotics, including oxytetracycline and doxycycline, accounted for 75% of all antibacterial substances determined in natural fertilizers.

Our study results are consistent with studies by other scientists that detection rates and concentrations of antibiotics tend to be higher in swine fertilizers than in poultry and cattle fertilizers. This is mainly due to the fact that antibiotics are administered in higher doses and more frequently to pigs than to other farm animals (Xin et al. 2016). In a study conducted in the Netherlands on 680 feces samples from 20 pig farms and 20 cattle farms, the presence of antibiotics was found, respectively, in 55% and 75% of samples (Berendsen et al. 2015). Moreover, more than one antibiotic was detected in as many as 34% of the tested samples. The most frequently detected compounds were oxytetracycline, doxycycline and sulfadiazine, tetracycline, flumequine, lincomycin, and tylosin. The antibacterial substances in the samples ranged from 1 to 95 mg/kg of feces (Berendsen et al. 2015). Similar results were obtained in this study, showing that the most common antibiotics in the analyzed fertilizers were tetracyclines, mainly doxycycline, and the determined contents of antibacterial substances were similar to those described by Berendsen et al. (2015). In the research conducted by Zhao et al. (2010), 143 samples of feces from eight Chinese provinces were analyzed, in which the presence of ciprofloxacin, enrofloxacin, oxytetracycline, and chlortetracycline in pig and cattle feces at concentrations ranging from 21 to over 59 mg/kg. No significant concentrations of sulfonamides (below 10 mg/kg) were found in any of the analyzed feces samples and only sulfadimidine was observed in chicken droppings at a maximum concentration of 6.04 mg/kg. The residues found by the authors for most antibiotics showed significant statistical differences between the provinces from which they were obtained samples collected and animal species (Zhao et al. 2010).

Laboratory analysis of commercial organic fertilizers also confirmed the presence of antibacterial substances. The determined levels of antibacterial substances in organic fertilizers were lower in relation to the concentrations of antibacterial substances determined in natural fertilizers and ranged from 47.0 to 757.9 µg/kg, but the results obtained may indicate that the applied processing processes such as increase in the temperature during the processing or composting do not lead to the complete degradation of antibacterial substances present in the material used. The tests carried out showed the presence of antibacterial substances in 9 out of 24 analyzed samples of commercial organic fertilizers made from manure (cattle, horse, or sheep), which constitutes over 37.5% of the positive results obtained. In solid commercial organic fertilizers, the most frequently found antibiotics were not only oxytetracycline and epi-oxytetracycline but also tiamulin, flumequine, sulfamethazine, sulfamerazine, sulfadiazine, lincomycin, and trimethoprim. Moreover, due to the fact that commercial organic fertilizers are produced from animal excrement from different farms, the presence of up to five different antibiotics was found in one fertilizer sample. In the case of detection and quantification of antibiotics in solid commercial organic fertilizers produced with animal by-products, there is practically no literature data on their analysis and the presence of antibiotics in them.

Based on the results obtained in this work and those of other researchers, it should be concluded that antibiotics are often present in natural and organic fertilizers that are used on agricultural fields and grasslands, which may pose a threat to the natural environment. The presence of veterinary antibiotics in natural and organic fertilizers should be monitored to ensure the safety not only of the environment but also of animals and consumers consuming agricultural produce and food of animal origin. Moreover, the European Parliament Resolution of June 1, 2023, on EU actions to combat antimicrobial resistance adopted by the European Union states that the use of sewage sludge and manure as fertilizers on agricultural soil may lead to the development of antimicrobial resistance through the spread of antimicrobial-resistant bacteria and antimicrobial resistance genes in the environment, which causes further contamination of the food chain and it is necessary to introduce prudent manure management practices. The results for positive samples of solid natural and organic fertilizers are summarized in Table 4.

**Table 4:** Results for positive samples of solid natural and organic fertilizers

No	Analyte [µg/kg]										
	Analyzed samples	OXT + epi-OXT	SMR	DC	SMX	TIAM	FLU	TRIM	SMZ	SDZ	LINCO
Natural fertilizers											
	Pig manure			189.2							
	Pig manure			178.6							
	Pig manure	1432.0 + 110.1		4048.2							
	Pig manure				10,874.2						
	Pig manure			956.1							
	Pig manure			1876.8							
	Pig manure			1089.2							
	Pig manure				6877.4						
	Pig manure				143.2						
	Pig manure			57,000.0							
	Pig manure			1120.8							
	Pig manure			57,750.0							
	Pig manure			55,830.0							
	Pig manure	94.5		3634.0		770.2					
	Pig manure	1272.0 + 52.0		32,020.0		3656.0					
	Pig manure			500.6							
	Pig manure			104.5							
	Pig manure			103.0							
	Poultry droppings				1987.2						
Commercial organic fertilizers											
20	Granulated manure		52.5								
21	Granulated cattle manure	208.0 + 238.0				610.9			294.0		
22	Granulated cattle manure	74.5 + 78.0					47.0				
23	Granulated cattle manure	142.0 + 160.0					220.6	98.3	133.6	103.0	
24	Granulated sheep manure								69.0		
25	Granulated cattle manure										757.9
26	Granulated manure									65.0	
27	Granulated cattle manure	291.1 + 340.9									
28	Granulated horse manure	255.3 + 306.8									

## Conclusion

A sensitive and robust method for the determination of 21 antibacterial substances in natural and organic solid fertilizers has been developed. Sample preparation was performed using SPE followed by analysis using UHPLC-MS/MS. This is one of the few described methods that allows for the simultaneous analysis of antibacterial substances in solid natural fertilizers and solid organic fertilizers produced on the basis of animal by-products. The analytical range of the method for all compounds allows their determination in the concentration range from 50 to 1000 µg/kg of fertilizer. The developed method was used to analyze 73 samples of solid natural and organic fertilizers. Twenty-eight samples were tested positive for antimicrobial substances. Antibiotic contamination was higher in natural fertilizers than in organic fertilizers, with the highest concentration of antibiotics in fertilizers from pigs. Moreover, the analysis results obtained for organic fertilizers showed that the processing processes used in their production do not result in complete degradation of antibiotics. The method described could be employed as a tool for monitoring the presence and persistence of antimicrobials in solid fertilizers.

## Data Availability

All relevant data has been included in the manuscript, and if more data is required, it will be made available on request.

## Abbreviations

CCα: Decision limit
CCβ: Detection capability
CIP: Ciprofloxacin
CTC: Chlortetracycline
CV: Coefficient of variation
DC: Doxycycline
DM: Dry matter
DMC: Demeclocycline
ENR: Enrofloxacin
epi-CTC: Epi-chlortetarcycline
epi-OXT: Epi-oxytetracycline
ERM: Erythromycin
ESI: Electrospray ionization
FLU: Flumequine
FQs: Fluoroquinolones
HPLC: High performance liquide chromatography
IS: Internal standard
LINCO: Lincomycin
LINCO-d3: Lincomycin-d3
LOD: Limit of detection
LOQ: Limit of quantification
MAs: Macrolides
MRM: Multiple reaction monitoring
MS: Mas spectrometry
MS/MS: Tandem mass spectrometry
NOR: Norfloxacin
OXT: Oxytetracycline
PLMs: Pleuromutillins
R^2^: Regression coefficient
SAR: Sarfloxacin
SAs: Sulfonamides
SD: Standard deviation
SDZ: Sulfadiazine
SDZ-^13^C_6_: Sulfadiazine-^13^C_6_
SGD: Sulfaguanidine
SMR: Sulfamerazine
SMT: Sulfamethazine
SMX: Sulfamethoxazole
SPE: Solid liquid extraction
SPIR: Spiramycin
TC: Tetracycline
TCs: Tetarcycylines
TIAM: Tiamulin
TRIM: Trimethoprim
TYL: Tylosin
U: Uncertainty
UHPLC-MS/MS: Ultra-high performance liquid chromatography tandem mass spectrometry
VAL: Valnemulin
VAs: Veterinary antibiotics
